# Difference between Anterior and Posterior Cord Compression and Its Clinical Implication in Patients with Degenerative Cervical Myelopathy †

**DOI:** 10.3390/jcm12124111

**Published:** 2023-06-18

**Authors:** Kyung-Chung Kang, Tae Su Jang, Sun-Hwan Choi, Hee-Won Kim

**Affiliations:** Department of Orthopaedic Surgery, Kyung Hee University Hospital, Kyung Hee University School of Medicine, Kyungheedae-ro 23, Dongdaemun-gu, Seoul 130-872, Republic of Korea; nolja86@hanmail.net (T.S.J.); swan8131@gmail.com (S.-H.C.); khmd48@naver.com (H.-W.K.)

**Keywords:** degenerative cervical myelopathy, anterior compression of spinal cord, anteroposterior compression ratio

## Abstract

In degenerative cervical myelopathy (DCM), the low anteroposterior compression ratio of the spinal cord is known to be associated with a neurologic deficit. However, there is little detailed analysis of spinal cord compression. Axial magnetic resonance images of 183 DCM patients at normal C2–C3 and maximal cord compression segments were analyzed. The anterior (A), posterior (P), and anteroposterior length and width (W) of the spinal cord were measured. Correlation analyses between radiographic parameters and each section of Japanese Orthopedic Association (JOA) scores and comparisons of the patients divided by A (below or above 0, 1, or 2 mm) were performed. Between C2–C3 and maximal compression segments, the mean differences of A and P were 2.0 (1.2) and 0.2 (0.8) mm. The mean anteroposterior compression ratios were 0.58 (0.13) at C2–C3 and 0.32 (0.17) at maximal compression. The A and A/W ratio were significantly correlated with four sections and the total JOA scores (*p* < 0.05), but the P and P/W ratio did not demonstrate any correlations. Patients with A < 1 mm had significantly lower JOA scores than those with A ≥ 1 mm. In patients with DCM, spinal cord compression occurs mainly in the anterior part and the anterior cord length of <1 mm is particularly associated with neurologic deficits.

## 1. Introduction

Degenerative cervical myelopathy (DCM) is mainly characterized by motor dysfunctions, such as hand clumsiness, gait disturbance, and/or bladder and bowel dysfunction [1]. In general, the severity of these neurologic deficits is known to be associated with a low anteroposterior (A-P) compression ratio or flattening shape of the spinal cord.

Previous studies have quantified the degree of cord compression in patients with DCM. Houser et al. reported that cervical myelopathy was highly correlated with a banana-shaped spinal cord [2], and Ono et al. measured the A-P and lateral diameters of the spinal cord using the actual spinal cord cross-section and calculated the ratio between the two. They concluded that patients with an A-P compression ratio of <0.4 had a worse prognosis [3]. Meanwhile, Ogino et al. revealed that an A-P compression ratio of ≥0.4 or a large cross-sectional area of ≥40 mm^2^ of the spinal cord would be good prognostic factors in patients with cervical myelopathy [4]. Until now, in DCM patients, the degree of spinal cord compression has been evaluated in the anteroposterior/width compression ratio or cross-sectional area of the spinal cord in axial magnetic resonance images.

From a clinical perspective, however, a protruded disc, degenerative osteophyte, or mass of ossification of the posterior longitudinal ligament mainly compresses the anterior part of the spinal cord and, moreover, patients’ kyphotic alignment can aggravate anterior spinal cord compression [5]. Anatomically, the anterior portion of the spinal cord plays an important role in the motor function of patients because the anterior part of the spinal cord contains the anterior horn, ascending and descending tracts and anterior spinal artery [6,7]. The anterior horn, which is directly connected to the anterior nerve root, transmits motor information to the target muscle and can cause muscle degeneration and weakness when damaged. Additionally, the spinal cord comprises ascending and descending tracks in charge of voluntary movement and pain/temperature sensation, respectively. The anterior spinal artery runs along the spinal cord and supplies blood to the anterior two-thirds of the spinal cord, including the ascending and descending tracks [8]. As such, anterior compression of the spinal cord is highly related to the patient’s neurologic deficits. Meanwhile, the posterior part of the cervical spinal cord is responsible for processing and transmitting sensory information related to touch, pressure sense, and proprioception. It plays a definite role in relaying sensory signals to the brain for further processing, recognizing shapes and objects by touch, and controlling the proper position and movement of the body [9,10]. Unlike the anterior part of the spinal cord, isolated posterior cord compression is rare. Known etiologies that mainly cause damage to the posterior spinal cord are ossification of the ligament flavum, posterior element infection, cystic or degenerative change of facet joints, etc.

Therefore, because the roles and pathologies of the anterior and posterior parts of the spinal cord are different, in addition to the research for the conventional anteroposterior compression ratio or elongated shape of the spinal cord, it is necessary to make detailed analyses for each anterior and posterior cord compression, respectively. To date, there is little research like this. We hypothesized that anterior cord compression is more important than posterior cord compression. This study aims to elucidate the difference between anterior and posterior cord compression and its clinical implication in patients with degenerative cervical myelopathy.

## 2. Materials and Methods

### 2.1. Study Design

We retrospectively reviewed medical records and radiographic findings of 211 patients who had undergone decompressive surgeries for a diagnosis of DCM between May 2016 and November 2020 at our institution. All patients who had pre-operative Japanese Orthopaedic Association (JOA) scores and magnetic resonance imaging taken at our institution were included in this study. Among them, 28 patients with a history of previous cervical surgery, fracture, trauma, infection, and inflammatory diseases and patients with cord compression at the C2−C3 segment were excluded.

### 2.2. Radiographic and Clinical Assessments

**Figure 1:**
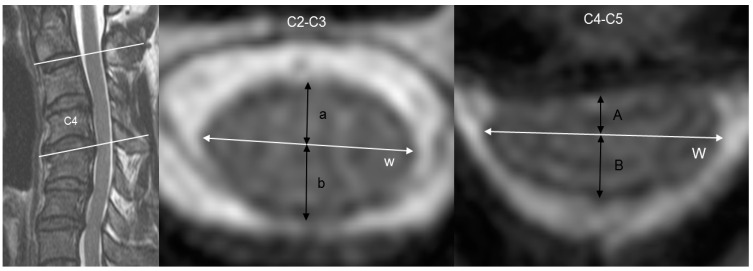
Cervical magnetic resonance images of a 49-year-old male patient with cervical spondylotic myelopathy and kyphotic alignment. He complained of severe hand clumsiness and whole-body numbness. His total Japanese Orthopaedic Association (JOA) score was 12 (I-0, II-4, III-5 and IV-3, respectively). The measured values were as follows: 3.7 mm (a), 3.9 mm (b), 12.9 mm (w), 1.8 mm (A), 3.7 mm (B) and 14.6 mm, respectively (W). The anterior length of the spinal cord decreased from 3.7 mm to 1.8 mm, but the posterior length slightly decreased from 3.9 mm to 3.7 mm.

The patients’ axial magnetic resonance images were selected from the horizontal centerline of the disc at the C2–C3 and maximal cord compression segment and compared. Anterior, posterior, anteroposterior and transverse lengths of the spinal cord were measured separately at the C2–C3 and maximal cord compression segments (Figure 1).

The anteroposterior length was defined as the diameter that started with the anterior median fissure and ended with the posterior median sulcus and the horizontal line from the outermost end to the opposite outer end. The anterior and posterior parts of the vertical line, which are divided by the horizontal line, are defined as the anterior and posterior length, respectively. To minimize inter- and intra-observer errors, two independent orthopedic surgeons who were blinded to clinical data evaluated the digital radiographs, which were uniformly magnified seven or eight times. The average measurements of two observers were used in this study. Inter- and intra-observer intra-class correlation coefficients (ICCs) were assessed for anteroposterior and transverse cord lengths.

The severity of patients’ symptoms of cervical myelopathy was evaluated using the JOA score, and the neurologic deficits of the patients were assessed [11,12]. The JOA score consists of 6 domains: motor function in upper extremities, motor function in lower extremities, and sensory function in upper extremities, trunk, and lower extremities, and bladder function, with a minimum total score of 0 and maximum of 17 (Table 1). Clinical assessment was performed using each of the four sections (upper extremity function (UEF, range: 0–4), lower extremity function (LEF, range: 0–4), sensory (range: 0–6), and bladder function (range: 0–3)) and the total JOA score (range: 0–17).

To elucidate the clinical implications of anterior and posterior cord compression, the anterior and posterior lengths, anteroposterior length, and transverse length of the spinal cord at the C2–C3 segment were compared with those at the maximal cord compression segment, and correlation analyses among the anterior cord length, posterior cord length, entire anteroposterior length, and each section and total JOA score were performed. At the maximal compression segment, the anterior/transverse length ratio, posterior/transverse length ratio, and anteroposterior/transverse ratio were calculated, and their correlations with the JOA score were analyzed. Additionally, to determine the reference point of anterior compression of the spinal cord, the patients were divided into two groups according to the anterior cord diameter (0 mm, 1 mm, and 2 mm), and the JOA (each section and total) scores were compared between the groups below and above the reference point.

### 2.3. Statistical Analysis

Statistical analysis was performed by a professional medical statistical consultant using SPSS version 19.0 statistical software (IBM Corp, Armonk, NY, USA). Values were presented as mean  ±  standard deviation. Depending on the normality of the data, correlations among the measured variables were analyzed using Pearson’s product-moment or Spearman’s rank correlation coefficient. An independent-sample *t*-test or Mann–Whitney *U*-test was used to compare the parameters. Significance was accepted for a *p*-value of <0.05.

### 2.4. Ethical Consideration and Approval

This study was approved by the Institutional Review Boards of Kyung Hee University Hospital and patients’ informed consent was waived owing to the retrospective design of the study (KHUH 2017-05-077-003, approved on 13 April 2020). All patients’ data were anonymized and kept confidential. All procedures were performed in compliance with the standards of our department and the Declaration of Helsinki.

## 3. Results

After applying the exclusion criteria, a total of 183 patients (123 men and 60 women, with a mean age of 59.5 ± 11.6 years) were included in this study. The patients’ diagnoses are cervical spondylotic myelopathy and herniated intervertebral disc (*n* = 115) and ossification of the posterior longitudinal ligament (*n* = 68). The mean total JOA score is 13.1 ± 3.2 and the mean of each section of the JOA score is 2.5 ±1.1 (JOA-UEF), 3.1 ± 1.2 (JOA-LEF), 4.8 ± 1.3 (sensory), and 2.7 ± 0.6 (bladder function). Inter- and intra-observer reproducibility was high for anteroposterior and transverse cord length measurement. The inter-observer ICCs for the anteroposterior and transverse length were 0.810 and 0.843 with corresponding intra-observer ICCs of 0.876 and 0.901, respectively.

At the C2–C3 disc segment, the mean anterior, posterior and anteroposterior cord lengths were 3.1 ± 0.6 mm, 4.3 ± 0.5 mm and 7.4 ± 1.0 mm, respectively, and the mean transverse length was 12.4 ± 1.2 mm. At the maximal compression level, the mean anterior, posterior and anteroposterior cord lengths were 1.0 ± 1.0 mm, 4.2 ± 0.8 mm and 5.3 ± 1.4 mm, respectively, and the mean transverse length was 14.2 ± 1.9 mm. Between the C2–C3 and maximal compression segments, decrements of the anterior and posterior cord lengths were 2.0 ± 1.2 mm and 0.1 ± 0.8 mm, respectively. The mean A-P compression ratios were 0.6 ± 0.1 and 0.3 ± 0.2 at C2–C3 and maximal compression segments, respectively (Table 2).

### 3.1. Correlation Analyses between Radiographic Parameters and JOA Score

The mean JOA score of the patients was 12.3 ± 4.8 (UEF: 2.8 ± 1.4, LEF: 3.1 ± 1.2, sensory: 5.0 ± 1.3 and bladder function: 2.7 ± 0.7). The anterior cord length was significantly correlated with all JOA scores as follows: total JOA (r = 0.254, *p* < 0.001), JOA-UEF (r = 0.212, *p* < 0.001), JOA-LEF (r = 0.182, *p* < 0.001), JOA-sensory (r = 0.148, *p* = 0.010) and JOA-bladder function (r = 0.149, *p* = 0.028). However, the posterior cord length did not show any correlations with the total JOA and each of the four sections. Meanwhile, the anteroposterior/transverse cord length ratio indicated significant correlations with the total JOA (r = 0.232, *p* < 0.001), JOA-UEF (r = 0.217, *p* < 0.001), JOA-LEF (r = 0.146, *p* = 0.044), and JOA-sensory (r = 0.163, *p* = 0.014), but not with JOA-bladder function (r = 0.094, *p* = 0.349). The anterior/transverse cord length ratio was significantly correlated with all JOA scores as follows: total JOA (r = 0.245, *p* < 0.001), JOA-UEF (r = 0.213, *p* < 0.001), JOA-LEF (r = 0.172, *p* = 0.008), JOA-sensory (r = 0.151, *p* = 0.012), and JOA-bladder function (r = 0.139, *p* = 0.019). However, the posterior/transverse cord length ratio did not indicate any significant correlations with the total JOA and each of the four sections (*p* > 0.05) (Table 3).

### 3.2. Reference Point for ACS

**Figure 2:**
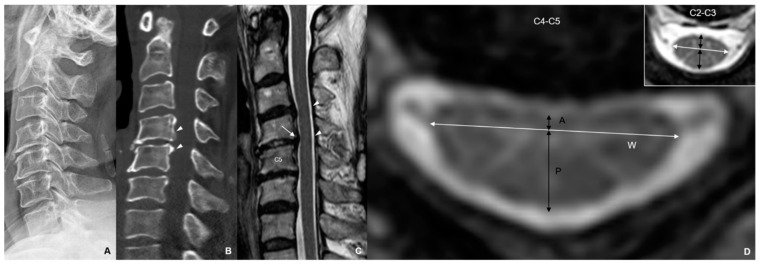
Radiographic images of a 56-year-old female patient with hand clumsiness and gait disturbance. Her total Japapanese Orthopadic Association (JOA) score was 13 (UEF-2, LEF-3, sensory-5, and bladder function-3). (**A**) Cervical lateral X-ray with normal alignment (at neutral position); (**B**) segmental ossification of posterior longitudinal ligament was shown at C4 and C5; (**C**) anterior compression of spinal cord was shown at C4−C5 segment (white arrow), but sufficient space behind the spinal cord was observed (arrow heads). (**D**) At C4−C5 segment, anterior (A) and posterior (P) lengths of the spinal cord were 0.85 and 4.84 mm, respectively, and anteroposterior (A-P)/width (W) ratio was 0.38. Anterior and posterior lengths of the spinal cord at C2−C3 were 2.56 and 4.84 mm, respectively, and AP/W ratio was 0.59. At 2 years after laminoplasty, her total JOA score improved from 13 to 16 (UEF-4, LEF-3, sensory-6, and bladder function-3) (UEF: upper extremity function); LEF: lower extremity function).

When confirming the association with the JOA score by dividing the degree of ACS into 0 mm, 1 mm, and 2 mm standards, the groups divided by 0 mm demonstrated significant difference only in the JOA-bladder function score between the groups with an ACS of <0 mm and ≥0 mm; the groups divided by 2 mm demonstrated no significantly different results in all JOA scores. By contrast, in the groups divided by 1 mm, the JOA-UEF, -LEF, -sensory, -bladder function, and the total scores were significantly higher in the group with an anterior cord compression of ≥1 mm than in the group with <1 mm (Table 4 and Figure 2).

## 4. Discussion

The results of this study were in line with those of previous reports that a low anteroposterior cord compression ratio was associated with neurologic deficits and poor prognosis in patients with DCM. However, to our knowledge, there is no study regarding the difference between anterior and posterior cord compression and its clinical implication in patients with DCM. With our results, the authors confirmed that spinal cord compression mainly occurred in the anterior part of the spinal cord but not in the posterior part. The anterior compression of the spinal cord was more significantly associated with motor weakness, particularly in the upper extremity, than posterior cord compression. Although the correlation coefficient was low, the anterior cord length and anterior/transverse cord length ratio were significantly correlated with all four sections and the total JOA score. By contrast, the posterior cord length and posterior/transverse cord length ratio did not indicate any correlations with the JOA scores. After confirming that anterior compression of the spinal cord is associated with neurologic deterioration, the patients were sub-grouped according to whether their anterior cord length was below and above 0 mm, 1 mm and 2 mm to determine the reference point of anterior compression of the spinal cord. Patients with an anterior cord length of <1 mm had significantly lower scores in each of the four sections and the total JOA score than patients with an anterior cord length of ≥1 mm.

Anterior compression of the spinal cord may play a more important role in patients with motor weakness than posterior compression because anterior protruded disc and/or kyphotic alignment generally affects the anterior horn of the spinal cord, motor neurons, and anterior spinal artery. The anterior horn, which is directly connected to the anterior nerve root, transmits motor information to the target muscle and can cause muscle degeneration and weakness when damaged [6,7,13]. Additionally, the anterior spinal artery runs along the spinal cord and supplies blood to the anterior two-thirds of the spinal cord, including the ascending and descending tracks [7]. Of the two tracts, the descending corticospinal tract comprises anterior and lateral tracks. As a major spinal pathway related to voluntary movement, it controls the primary motor activity of the somatic motor system [14]. Meanwhile, the ascending spinothalamic tract is a sensory tract that consists of an anterior tract responsible for sensory input to crude touch and a lateral tract that transmits information on pain and temperature sensation [15]. For all these reasons, anterior compression of the spinal cord is highly related to the patient’s neurologic symptoms.

However, to date, only a few reports have emphasized the importance of anterior cord compression. In 2012, Hirai et al. reported the decompression effect of laminoplasty as both a direct posterior and indirect anterior decompression effect. The existence of anterior compression of the spinal cord after laminoplasty may adversely affect postoperative clinical outcomes and interfere with the improvement of upper extremity motor function in patients with myelopathy [16]. However, they only demonstrated the presence of anterior cord compression and did not evaluate the degree of anterior cord compression and its clinical relevance. Sodeyama et al. reported that an indirect anterior decompression with ≥3 mm shift of the posterior spinal cord induces satisfactory relief of myelopathy symptoms [17]. This study also emphasized the clinical significance of anterior cord compression in patients with DCM, although it did not reveal a direct association between anterior cord compression and patients’ neurologic deficits.

Degenerative cervical myelopathy can show various symptoms and signs depending on the severity and location of spinal cord compression, and it is sometimes difficult to distinguish it from other diseases [7,13]. Damage to the anterior horn of the spinal cord can lead to a clinical presentation that mimics some features of amyotrophic lateral sclerosis (ALS) [18]. The anterior horns contain motor neurons transmitting the signals from the spinal cord to the muscles. Therefore, anterior cord compression may affect the patient’s muscle weakness, atrophy, and fasciculations. These symptoms are often similar to those of ALS. Meanwhile, posterior columns of the spinal cord are involved with touch, proprioception, and vibration. Damage to the posterior columns in cervical myelopathy can result in posterior column ataxia. Clinical manifestations may include a loss of proprioception, impaired touch discrimination, and difficulties with balance. If anterior or lateral corticospinal tracts in the spinal cord are compressed, it would affect voluntary movement and fine motor control. The patients may reveal muscle weakness and spasticity as well as hyperreflexia in the upper and lower extremities. The compression of the sensory pathway may cause loss of position sense, diminished tactile sensation, and impaired vibration sense [14]. As such, different symptoms may appear depending on the location and degree of spinal cord compression; therefore, proper diagnosis and assessment are necessary to accurately identify specific neurologic deficits. To date, most of the previous studies have not assessed the extent of neurological symptoms and signs that vary depending on the compression areas of the spinal cord. In the results of our study, the authors could have concluded that the main compression of the spinal cord occurred in the anterior part, not posterior part, in patients with degenerative cervical myelopathy, and anterior compression has revealed more significant neurologic deficits than the posterior cord compression. In the future, it is necessary to develop a measurement method that can measure the detailed compression location of the spinal cord. Interestingly, from a clinical perspective, some patients demonstrate isolated anterior compression of the spinal cord without a low anteroposterior compression ratio. In such cases, if the patients complain of neurologic symptoms, surgeons cannot easily determine whether anterior compression of the spinal cord is associated with the patients’ neurologic deficits. In the present study, to differentiate the reference point of meaningful anterior compression of the spinal cord, the authors sub-grouped the patients according to the anterior cord lengths of 0 mm, 1 mm and 2 mm at the maximal cord compression segments and conducted a comparison analysis between the groups. In the results, the anterior cord length of 1 mm was determined as the reference point for neurologic abnormalities in patients with DCM. According to our results, an anterior cord length of <1 mm would likely be associated with neurologic deterioration, even in patients with sufficient space at the posterior portion of the spinal cord or a high A-P/W compression ratio. Therefore, if patients with an anterior cord length of <1 mm and significant motor weakness undergo cervical spinal surgeries, anterior decompression surgery during the operation should be performed in order to achieve good surgical outcomes.

Although it was not investigated in this study, recently, Banerjee et al. revealed the association between the prevalence of DCM and pathologic type on magnetic resonance imaging and emphasized understanding the natural history of DCM [19]. Some studies have revealed that present pathobiological and mechanical knowledge does not explain the severity of DCM. On the contrary, they emphasized that the pattern of mechanical stress, the patient’s factor for vulnerability and a relevant time are important for patients’ clinical symptoms [20]. Some researchers reported the value of quantitative assessment, such as microstructure, perfusion, and electrophysiology techniques [21]. Along with these current studies, the authors thought that a proposed new technique that can measure anterior spinal cord compression would be helpful to explain a gap between the imaging findings and the actual clinical symptoms.

This study has several limitations. First, because this study was conducted based on detailed image analysis, the radiographic measurements may have some errors. To reduce such errors, the images that were magnified over seven or eight times were measured twice by two orthopedic surgeons, and inter- and intra-observer ICCs were assessed. Second, in our results, although the correlations between the parameters for anterior cord compression and the JOA scores were significant, such correlations seemed to be weak. Last, analyses for the subtype of patients’ cord compression (e.g., anterior, posterior or circumferential) and locations of spinal cord damage were not performed. In the future, it seems necessary to study these contents. Nevertheless, in this study, the anterior cord length demonstrated similar or more significant correlations with all the JOA scores than the previous anteroposterior compression ratio, and the patients with an anterior cord length of <1 mm revealed a worse clinical prognosis. Moreover, the results of this study are considered to be meaningful because studies regarding the anterior compression of the spinal cord and its clinical significance are quite rare.

## 5. Conclusions

In patients with DCM, a low anteroposterior compression ratio was significantly correlated with neurologic deficits, but spinal cord compression occurred mainly in the anterior part of the spinal cord. Anatomically, the anterior part of the spinal cord is likely to be directly related to the patient’s neurological function, rather than the posterior part of the spinal cord. Therefore, anterior compression of the spinal cord was more significantly associated with neurologic abnormality than posterior compression. In this study, an anterior cord length of <1 mm is associated with significant neurologic deficits.

## Figures and Tables

**Table 1:** Japanese Orthopaedic Association (JOA) score.

**1. Upper Extremity Motor Function**		**2. Lower Extremity Motor Function**	
**0**	Impossible to eat with chopsticks or spoon	**0**	Cannot walk
**1**	Possible to eat with spoon but not with chopsticks	**1**	Needs cane or aid on flat ground
**2**	Possible to eat with chopsticks, but to a limited degree	**2**	Needs cane or aid only on stairs
**3**	Possible to eat with chopsticks, awkward	**3**	Can walk without cane or aid but slowly
**4**	No disability	**4**	No disability
**3. Sensory Function**		**4. Bladder Function**	
**A. Upper extremity**		**0**	Complete retention
**0**	Apparent sensory loss	**1**	Severe disturbance
**1**	Minimal sensory loss	**2**	Mild disturbance
**2**	Normal	**3**	Normal
**B. Trunk (same as A)**			
**C. Lower extremity (same as A)**			

**Table 2:** Comparison and change in the anterior, posterior, anteroposterior and transverse cord lengths between the C2–C3 (normal) and maximal cord compression segments (Cmax: length at the maximal cord compression segment).

Length (mm)	C23 (Mean)	Maximal Cord Compression (Mean)	Difference (C23-Cmax)	*p*-Value
Anterior	3.1 ± 0.6	1.0 ± 1.0	2.0 ± 1.2	<0.001 *
Posterior	4.3 ± 0.54	4.2 ± 0.8	0.1 ± 0.8	0.187
Anteroposterior (A-P)	7.4 ± 1.0	5.3 ± 1.4	2.1 ± 0.5	<0.001 *
Width	12.4 ± 1.2	14.2 ± 1.9	−1.8 ± 0.2	<0.001 *
A-P/Width	0.6 ± 0.1	0.3 ± 0.2	0.3 ± 0.1	<0.001 *

*: *p* < 0.05.

**Table 3:** At the segment of the maximal cord compression, correlation analyses among several parameters and each of the four sections as well as the total Japanese Orthopaedic Association (JOA) score were demonstrated. In the results, A, A + P/W and A/W revealed significant correlations with most of the JOA scores, whereas P and P/W were not associated with any sections of the JOA score. (A: anterior, P: posterior, W: width, UEF: upper extremity function, and LEF: lower extremity function).

Correlation Coefficient	JOA-UEF	JOA-LEF	JOA-Sensory	JOA-Bladder Function	JOA-Total
A	0.212 **	0.182 **	0.148 *	0.149 *	0.254 **
P	−0.028	0.010	−0.011	−0.037	−0.008
A + P/W	0.217 **	0.146 *	0.163 *	0.094	0.232 **
A/W	0.213 **	0.172 *	0.151 *	0.139 *	0.245 **
P/W	0.060	−0.020	0.015	−0.062	0.009

A: anterior length, P: posterior length, W: width (at the segment of maximum cord compression), **: *p* < 0.001, *: *p* < 0.05.

**Table 4:** Comparisons of the two groups divided by reference point of anterior compression of the spinal cord (anterior length: 0 mm, 1 mm or 2 mm). When the patients were sub-grouped by anterior cord length of 1 mm at the maximal compression segment, the patients with an anterior length of <1 mm had significantly lower scores in each of four sections and the total JOA scores than those with an anterior cord length of ≥1 mm (*p* < 0.05), but the differences were scarce when the patients were divided by the anterior cord length of 0 or 2 mm (UEF: upper extremity function, and LEF: lower extremity function).

Subgroups (Anterior Length)	Number of Patients	JOA-UEF	JOA-LEF	JOA-Sesory	JOA-Bladder Function	JOA Total
≥0 mm	145	2.6 ± 1.1	3.2 ± 1.2	4.9 ±1.3	2.7 ±0.5	13.4 ± 3.1
<0 mm	38	2.4 ± 1.1	2.9 ± 1.3	4.6 ± 1.4	2.5 ± 0.8	12.4 ± 3.4
*p*-value		0.475	0.090	0.252	0.039 *	0.083
≥1 mm	91	2.8 ± 1.1	3.4 ± 1.0	5.0 ± 1.2	2.8 ± 0.5	14.0 ± 2.9
<1 mm	92	2.3 ± 1.1	2.9± 1.3	4.6± 1.4	2.6 ± 0.7	12.4 ± 3.3
*p*-value		0.001 *	0.004 *	0.018 *	0.020 *	<0.001 *
≥2 mm	21	2.6 ± 1.0	3.4 ± 1.1	4.8 ± 1.3	2.8 ± 0.5	13.5 ± 3.2
<2 mm	162	2.5 ± 1.2	3.1 ± 1.2	4.8 ± 1.5	2.7 ± 0.6	13.1 ± 3.1
*p*-value		0.744	0.154	0.962	0.861	0.353

*: *p* < 0.05.

## Data Availability

All data derived from this study are presented in the text.

## References

[1] Paul A.J., Amritanand R., Margabandhu P., Karuppusami R., David K.S., Krishnan V. (2021). Composite Grip Strength as a Marker of Outcome in Patients Surgically Treated for Degenerative Cervical Myelopathy. Asian Spine J..

[2] Houser O.W., Onofrio B.M., Miller G.M., Folger W.N., Smith P.L. (1994). Cervical spondylotic stenosis and myelopathy: Evaluation with computed tomographic myelography. Mayo Clin. Proc..

[3] Ono K., Ota H., Tada K., Yamamoto T. (1977). Cervical Myelopathy Secondary to Multiple Spondylotic Protrusions: A Clinicopathologic Study. Spine.

[4] Ogino H., Tada K., Okada K., Yonenobu K., Yamamoto T., Ono K., Namiki H. (1983). Canal diameter, anteroposterior compression ratio, and spondylotic myelopathy of the cervical spine. Spine.

[5] Shimizu T., Fujibayashi S., Otsuki B., Murata K., Masuda S., Matsuda S. (2022). Residual anterior cord compression after laminoplasty for cervical spondylotic myelopathy: Evaluation of risk factors according to the most severely stenotic vertebral segment. J. Neurosurg. Spine.

[6] Diaz E., Morales H. (2016). Spinal Cord Anatomy and Clinical Syndromes. Semin. Ultrasound CT MR.

[7] Hardy T.A. (2021). Spinal Cord Anatomy and Localization. Continuum.

[8] Jacob I.L., Ringstad G.A., Enriquez B.A., Jusufovic M. (2023). Spinal artery infarction. Tidsskr. Nor. Laegeforen.

[9] Kieser D.C., Cox P.J., Kieser S.C.J. (2018). Hirayama disease. Eur. Spine J..

[10] Yoshida G., Ushirozako H., Hasegawa T., Yamato Y., Yasuda T., Banno T., Arima H., Oe S., Mihara Y., Yamada T. (2022). Selective Angiography to Detect Anterior Spinal Artery Stenosis in Thoracic Ossification of the Posterior Longitudinal Ligament. Asian Spine J..

[11] Yonenobu K., Abumi K., Nagata K., Taketomi E., Ueyama K. (2001). Interobserver and intraobserver reliability of the japanese orthopaedic association scoring system for evaluation of cervical compression myelopathy. Spine.

[12] Hirabayashi K., Miyakawa J., Satomi K., Maruyama T., Wakano K. (1981). Operative results and postoperative progression of ossification among patients with ossification of cervical posterior longitudinal ligament. Spine.

[13] Cho T.A. (2015). Spinal cord functional anatomy. Continuum.

[14] Van Wittenberghe I.C., Peterson D.C. (2022). Corticospinal Tract Lesion. StatPearls.

[15] Al-Chalabi M., Reddy V., Gupta S. (2022). Neuroanatomy, Spinothalamic Tract. StatPearls.

[16] Hirai T., Kawabata S., Enomoto M., Kato T., Tomizawa S., Sakai K., Yoshii T., Sakaki K., Shinomiya K., Okawa A. (2012). Presence of anterior compression of the spinal cord after laminoplasty inhibits upper extremity motor recovery in patients with cervical spondylotic myelopathy. Spine.

[17] Sodeyama T., Goto S., Mochizuki M., Takahashi J., Moriya H. (1999). Effect of decompression enlargement laminoplasty for posterior shifting of the spinal cord. Spine.

[18] Torrieri M.C., Monticelli M., Vasta R., Cofano F., Ajello M., Canosa A., Penner F., Marengo N., Manera U., Calvo A. (2020). Comorbidity of Cervical Spondylogenic Myelopathy and Amyotrophic Lateral Sclerosis: When Electromyography Makes the Difference in Diagnosis. Eur. Neurol..

[19] Banerjee A., Mowforth O.D., Nouri A., Budu A., Newcombe V., Kotter M.R.N., Davies B.M. (2022). The Prevalence of Degenerative Cervical Myelopathy-Related Pathologies on Magnetic Resonance Imaging in Healthy/Asymptomatic Individuals: A Meta-Analysis of Published Studies and Comparison to a Symptomatic Cohort. J. Clin. Neurosci..

[20] Davies B.M., Mowforth O., Gharooni A.A., Tetreault L., Nouri A., Dhillon R.S., Bednarik J., Martin A.R., Young A., Takahashi H. (2022). A New Framework for Investigating the Biological Basis of Degenerative Cervical Myelopathy [AO Spine RECODE-DCM Research Priority Number 5]: Mechanical Stress, Vulnerability and Time. Glob. Spine J..

[21] Martin A.R., Tetreault L., Nouri A., Curt A., Freund P., Rahimi-Movaghar V., Wilson J.R., Fehlings M.G., Kwon B.K., Harrop J.S. (2022). Imaging and Electrophysiology for Degenerative Cervical Myelopathy [AO Spine RECODE-DCM Research Priority Number 9]. Glob. Spine J..

